# Knockdown of insulin-like growth factor 2 gene disrupts mitochondrial functions in the liver

**DOI:** 10.1093/jmcb/mjab030

**Published:** 2021-05-14

**Authors:** Weiwei Gui, Yiyi Zhu, Shuiya Sun, Weifen Zhu, Bowen Tan, Hanxin Zhao, Chengxin Shang, Fenping Zheng, Xihua Lin, Hong Li

**Affiliations:** 1 Department of Endocrinology, The Affiliated Sir Run Run Shaw Hospital, School of Medicine, Zhejiang University, Hangzhou, China; 2 Department of Endocrinology, Peking Union Medical College Hospital, Peking Union Medical College, Chinese Academy of Medical Sciences, Beijing, China; 3 College of Medicine, Zhejiang University, Hangzhou, China; 4 Biomedical Research Center and Key Laboratory of Biotherapy of Zhejiang Province, The Affiliated Sir Run Run Shaw Hospital, Zhejiang University, Hangzhou, China

**Keywords:** insulin-like growth factor 2, mitochondrial function, nonalcoholic fatty liver

## Abstract

Even though insulin-like growth factor 2 (IGF2) has been reported to be overexpressed in nonalcoholic fatty liver disease (NAFLD), its role in the progression of NAFLD and the potential mechanism remain largely unclear. Using *in vitro* models, we found that IGF2 was the key overexpressed gene in steatosis, suggesting a possible association between IGF2 and NAFLD. Interestingly, loss-of-function experiments revealed that inhibition of IGF2 protein impaired mitochondrial biogenesis and respiration. It additionally disrupted the expression changes of mitochondrial fusion and fission-related proteins necessary in maintaining mitochondrial homeostasis. Consistently, IGF2 knockdown reduced the mitochondrial membrane potential and increased the production of reactive oxygen species. Mechanistically, IGF2 regulates mitochondrial functions by modulating the expression of SIRT1 and its downstream gene PGC1α. This research opens a new frontier on the role of IGF2 in energy metabolism, which potentially participates in the development of NAFLD. As such, IGF2 is a potential therapeutic target against NAFLD.

## Introduction

Insulin-like growth factor 2 (IGF2), which shares ∼67% homology with IGF1, is one of the most critical components in the IGF system. Previous studies have mainly focused on the physiological role of IGF1, with IGF2 thought to be the ‘second’ IGF, thus attracting substantially less interest when compared with IGF1 (Chao and D'Amore, 2008). However, there is growing evidence that IGF2 regulates fetal growth and development and as well participates in the development of numerous diseases such as cancer, obesity, diabetes, and liver diseases (Wang et al., 2003; Vella and Malaguarnera, 2018; Minchenko et al., 2019; Yu et al., 2019). Notably, the concentrations of serum IGF2 are three times of that of IGF1, underlining the physiological significance of IGF2 (Humbel, 1990).

As mentioned above, IGF2 participates in the development of chronic liver diseases. In particular, numerous studies have demonstrated the upregulated expression of IGF2 in nonalcoholic fatty liver disease (NAFLD) (Chiappini et al., 2006; Tybl et al., 2011; Kessler et al., 2016). Given its global distribution and high prevalence of 10%‒30%, NAFLD understandably attracts considerable interest (Loomba and Sanyal, 2013). NAFLD is characterized by excess lipid deposition in the liver, predisposing the liver to multiple damages ranging from simple steatosis to the end-stage liver disease hepatocellular carcinoma (HCC). Dysregulation of mitochondrial functions is believed to potentially disrupt normal cellular metabolic processes, aiding in the development of NAFLD. Chiappini et al. (2006) reported that normal mitochondrial functioning ensures optimal catabolism of hepatic lipids. However, steatosis disrupts mitochondrial oxidative metabolism (Chiappini et al., 2006). Yet, it is not clear whether IGF2 participates in the progression of NAFLD by damaging structural integrity and molecular functions of the mitochondria.

Recently, few studies have attempted to uncover the role of IGF2 in mitochondrial functions. For instance, Vella et al. (2019) reported that overexpression of IGF2 promoted both aerobic glycolysis and biogenesis in the mitochondria, which may lead to mutations in breast cancer cells. In the related research, Younis et al. (2020) found that ZBED6‒IGF2 regulates myogenesis by modulating mitochondrial functions. These researches suggest a critical role of IGF2 in the mitochondria during NAFLD progression.

In this study, we hypothesized that IGF2 is essential in maintaining mitochondrial homeostasis in the liver. Our findings demonstrated that IGF2 was significantly upregulated in free fatty acid-induced steatosis in HepG2 and AML12 cells. Bioinformatics analyses further revealed that IGF2 participates in hepatic lipid metabolism and regulation of mitochondrial functions. Consistently, IGF2 knockdown markedly disrupted mitochondrial functions. Particularly, IGF2 knockdown impaired mitochondrial respiration and biogenesis, disrupted the mitochondrial membrane potential (MMP), and increased the production of reactive oxygen species (ROS) in HepG2 and AML12 cells. Mechanistically, IGF2 regulates these functions by modulating the expression of mitochondrial function-related genes including PGC1α and SIRT1.

## Results

### IGF2 is overexpressed in free fatty acid (FFA)-treated liver cells and liver tissues of obese mice

**Figure 1:**
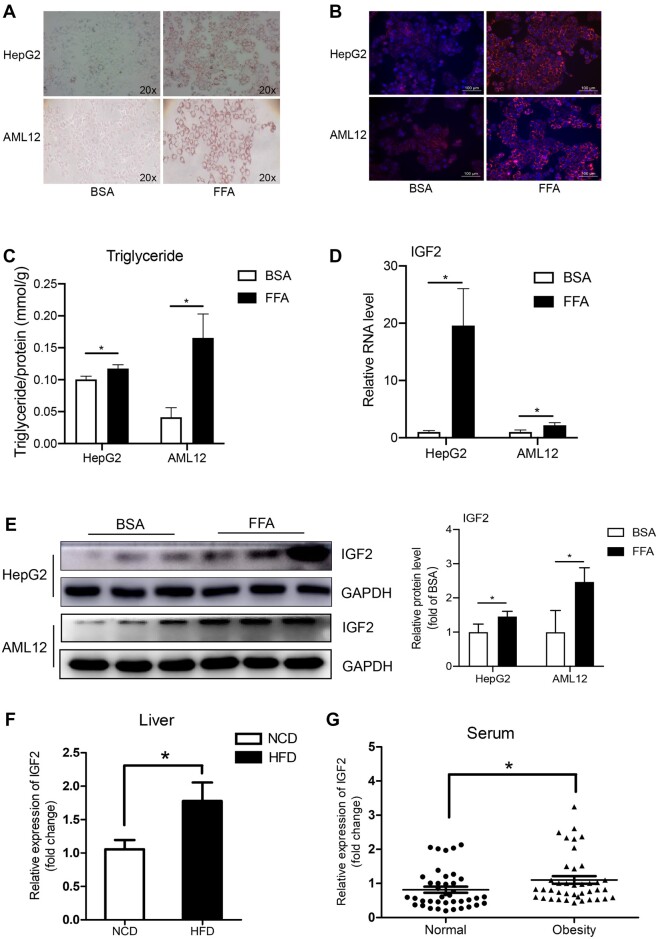
Expression of IGF2 in FFA-treated HepG2 and AML12 cells. (**A**) Representative images of FFA-induced steatosis in HepG2 and AML12 cells following Oil Red O staining. (**B**) Photos of FFA-induced steatosis in HepG2 and AML12 cells following Nile Red staining. Scale bar, 100 μm. (**C**) The expression of triglyceride in FFA-induced steatosis in HepG2 and AML12 cells. (**D**) RT−qPCR analysis for IGF2 mRNA expression in FFA-induced steatosis in HepG2 and AML12 cells. (**E**) Western blotting analysis for the expression of IGF2 protein in FFA-induced steatosis in HepG2 and AML12 cells. Representative gel images (left) and the quantitative data (right) are presented. (**F**) RT−qPCR analysis for IGF2 mRNA expression in HFD-induced obese mouse liver tissues (*n* = 5) and controls (*n* = 5). (**G**) RT−qPCR analysis for serum IGF2 levels in obese patients (*n* = 40) and controls (*n* = 40). The data are based on three independent experiments. Continuous data were expressed as mean ± SD. *P < 0.05.

Oil Red O and Nile red staining reveal that 0.75 mM FFA treatment effectively induced cellular lipid ectopic deposition in HepG2 and AML12 cells (Figure 1A and B). Similarly, FFA treatment increased the accumulation of triglyceride in HepG2 and AML12 cells (Figure 1C). Reverse transcription‒quantitative polymerase chain reaction (RT‒qPCR) and western blotting analyses further validated the upregulated expression of IGF2 mRNA and corresponding protein levels in HepG2 and AML12 cells. Combined, these findings demonstrate that FFA promotes the expression of IGF2 mRNA and production of corresponding proteins in HepG2 and AML12 cells (Figure 1D and E).

Meanwhile, we found that the levels of IGF2 mRNA in the liver tissues of high-fat diet (HFD)-fed mice were nearly 2-fold more than that of NCD mice (*P* < 0.05; Figure 1E). Further RT‒qPCR analysis validated the upregulated expression of IGF2 mRNA in obese mice (*P* < 0.05; Figure 1G). Thus, our findings suggest that IGF2 substantially regulates hepatic lipid metabolism.

### IGF2 regulates lipid metabolism and mitochondrial functions

Bioinformatic analysis of GSE116421 dataset uncovered 1757 differentially expressed genes (DEGs), 935 of which were upregulated whereas 822 were downregulated (Figure 2A and B) by IGF2 knockdown. Gene Ontology (GO) analysis revealed that the downregulated genes regulated metabolism of lipids such as fatty acids, steroid, and beta fatty acids, as well as catabolism of small molecules (Figure 2C). On the other hand, the upregulated genes mainly regulated mitochondrial-related pathways such carboxylic acid, glutamine family amino acid, and alpha-amino acid catabolism, multicellular organismal homeostasis, and sodium ion transport (Figure 2D). Kyoto Encyclopedia of Genes and Genomes (KEGG) analysis revealed comparable findings, in which the DEGs were linked to lipid metabolism and mitochondrial functions (Figure 2E and F). The GO plot of the DEGs is shown in Figure 2G.

**Figure:**
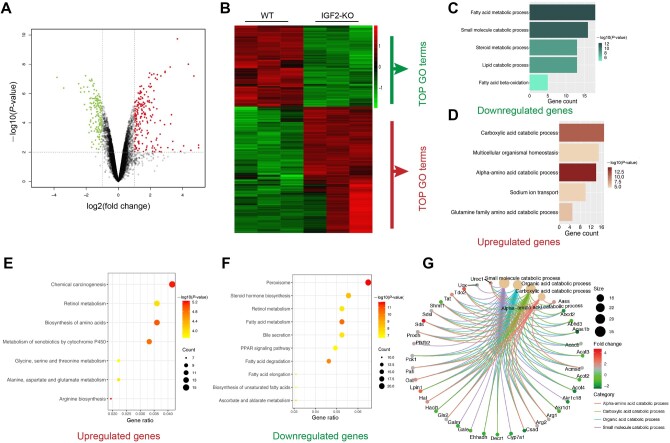
**Figure 2** Bioinformatics analyses for the relationship between IGF2 expression and hepatic lipid metabolism as well as mitochondrial functions. (**A** and **B**) Representative volcano plot (**A**) and heat map (**B**) of DEGs in the liver tissues of wide-type and IGF2 knockout mice. (**C** and **D**) GO analysis for the biological processes regulated by downregulated (**C**) and upregulated (**D**) genes. (**E** and **F**) KEGG pathway enrichment analysis of upregulated (**E**) and downregulated (**F**) DEGs. (**G**) Representative GO plot for biological processes regulated by DEGs.

RT‒qPCR was then used to confirm DEGs that were enriched in mitochondria-related pathways. Knockdown of IGF2 increased the expression levels of several genes enriched in ‘alpha-amino acid catabolic process’ pathway, such as OAT2, AASS, PRODH, TAT, ACMSD, and TOD2 in both HepG2 and AML12 cells. Interestingly, ‘alpha-amino acid catabolic process’-related gene SDHA was downregulated after knocking down IGF2 in HepG2 but not in AML12 cells. Moreover, PGC1β was decreased in AML12 cells after IGF2 depletion (Supplementary Figure S1). In summary, the results reveal a possible key role of IGF2 in lipid metabolism and mitochondrial functions.

### IGF2 knockdown in HepG2 and AML12 cells impairs mitochondrial functions

**Figure 3:**
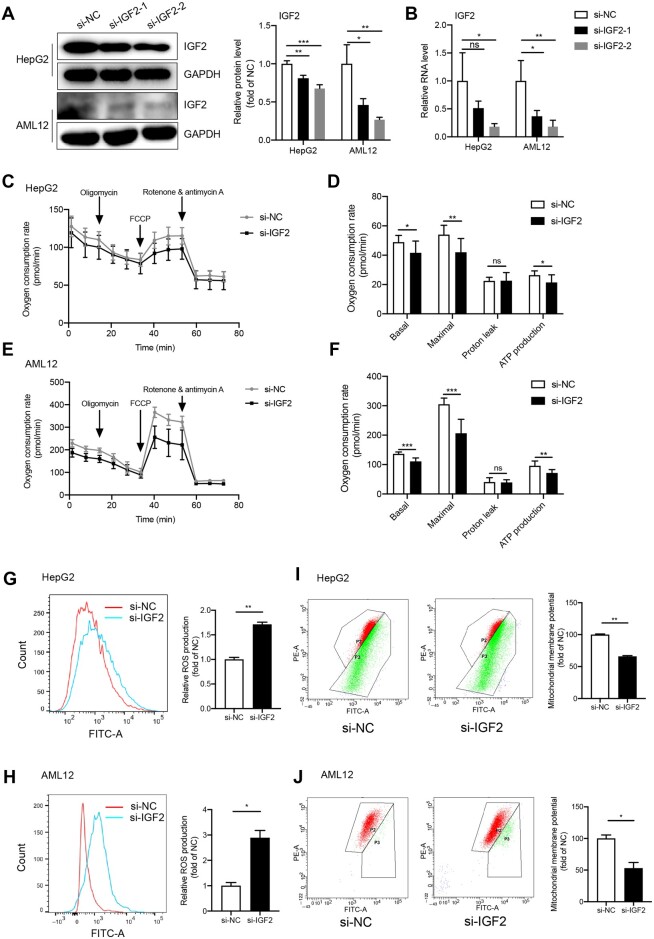
Effects of IGF2 knockdown in HepG2 and AML12 cells on mitochondrial functions. (**A**) Western blotting analysis for the expression of IGF2 protein in HepG2 and AML12 cells following si-IGF2 interference. Representative gel images (left) and the quantitative data (right) are presented. (**B**) RT−qPCR analysis for IGF2 mRNA expression in AML12 and HepG2 cells following si-IGF2 treatment. (**C** and **E**) Representative oxygen consumption rates in HepG2 (**C**) and AML12 (**E**) cells following IGF2 silencing. (**D** and **F**) Effects of IGF2 silencing on basal and maximal respiration, proton leakage, and ATP production in HepG2 (**D**) and AML12 (**F**) cells following IGF2 silencing. (**G** and **H**) The ROS production in HepG2 (**G**) and AML12 (**H**) cells following IGF2 inhibition. Representative images for ROS production based on flow cytometric analyses (left) and the quantified ROS production (right) are presented. (**I** and **J**) MMP in HepG2 (**I**) and AML12 (**J**) cells following IGF2 knockdown. Representative images of MMP based on flow cytometric test (left) and the quantified MMP (right) are presented. Results represent findings of three independent experiments. Continuous variables were expressed as mean ± SD. ***P < 0.001, **P < 0.01, *P < 0.05, ns, not significant.

Western blotting and RT‒qPCR revealed successful inhibition of IGF2 in HepG2 and AML12 cells using si-IGF2-2 (Figure 3A and B). Seahorse analysis revealed that IGF2 knockdown inhibited basal and maximal respiration in HepG2 and AML12 cells. Even though IGF2 knockdown disrupted ATP production in these cells, it had no effect on proton leak from the cells (Figure 3C‒F). In addition, IGF2 silencing induced overproduction of ROS in HepG2 and AML12 cells (Figure 3G and H). Similarly, IGF2 knockdown in HepG2 and AML12 cells decreased MMP (Figure 3I and J). Overall, IGF2 knockdown disrupts several mitochondrial functions.

### IGF2 knockdown disrupts mitochondrial biogenesis

**Figure 4:**
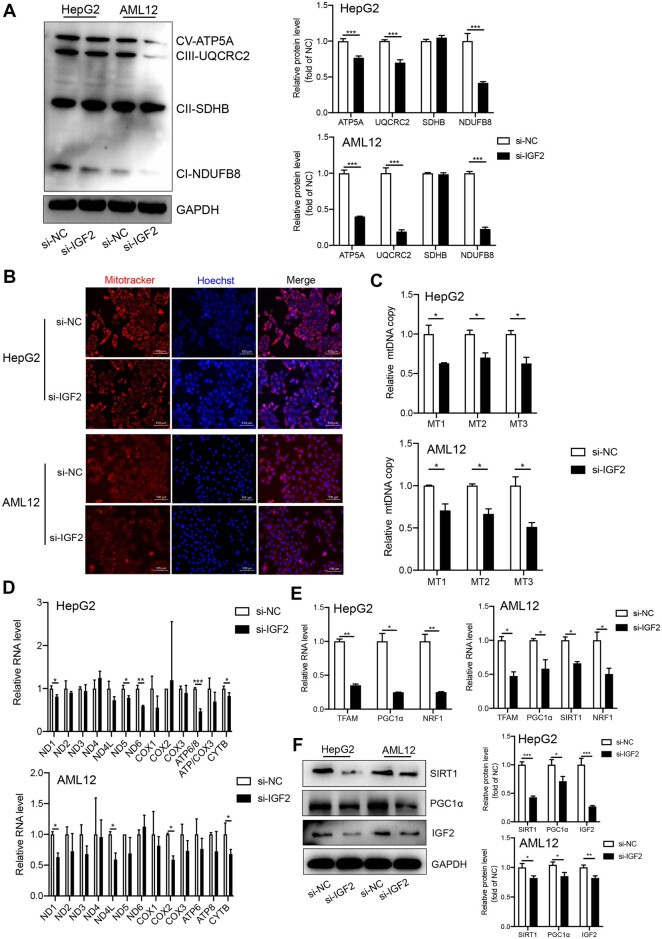
Effects of IGF2 knockdown on mitochondrial biogenesis. (**A**) Western blotting analysis for the expression of OXPHOS in HepG2 and AML12 cells following si-IGF2 pretreatment. Representative gel images (left) and the quantified data (right) are presented. (**B**) Representative images of si-IGF2-treaed HepG2 and AML12 cells following mitotracker staining. Scale bar, 100 μm. (**C**) RT−qPCR analysis for the relative mtDNA copy number in HepG2 (upper) and AML12 (lower) cells following si-IGF2 treatment. (**D**) RT−qPCR analysis for relative RNA levels of the mt-mRNAs in HepG2 (upper) and AML12 (lower) cells following si-IGF2 treatment. (**E**) RT−qPCR analysis for relative RNA levels of the mitochondrial transcription-related genes in HepG2 (left) and AML12 (right) cells following si-IGF2 treatment. (**F**) Western blotting analysis for protein levels of mitochondrial transcription-related genes in HepG2 (left) and AML12 (right) cells following si-IGF2 treatment. Results represent data for three independent experiments. Continuous variables were expressed as mean ± SD. ***P < 0.001, **P < 0.01, *P < 0.05.

Mitotracker staining of mitochondrial proteins and mRNA detection following IGF2 knockdown in HepG2 and AML2 cells revealed that IGF2 knockdown modulated the expression of mitochondrial electronic chain complex proteins (ATP5A, UQCRC2, and NDUFB8) but had no effects on the expression of SDHB (Figure 4A). In addition, IGF2 silencing reduced the numbers of mitochondria (Figure 4B) and mitochondrial DNA (mtDNA) copy (Figure 4C) in both HepG2 and AML12 cells. Meanwhile, RT‒qPCR for mtDNA-encoded mRNA (mt-mRNA) levels further validated the low mtDNA copy numbers in IGF2-deficient HepG2 and AML12 cells (Figure 4D).

RT‒qPCR analysis revealed that inhibition of IGF2 downregulated the expression of TFAM, PGC1α, and NRF1 mRNAs in HepG2 and AML12 cells (Figure 4E). Western blotting analysis further demonstrated the downregulated expression of SIRT1 and PGC1α proteins following IGF2 silencing (Figure 4F). Overall, these findings demonstrate that IGF2 knockdown impairs mitochondrial biogenesis.

### IGF2 knockdown promotes mitochondrial fission

**Figure 5:**
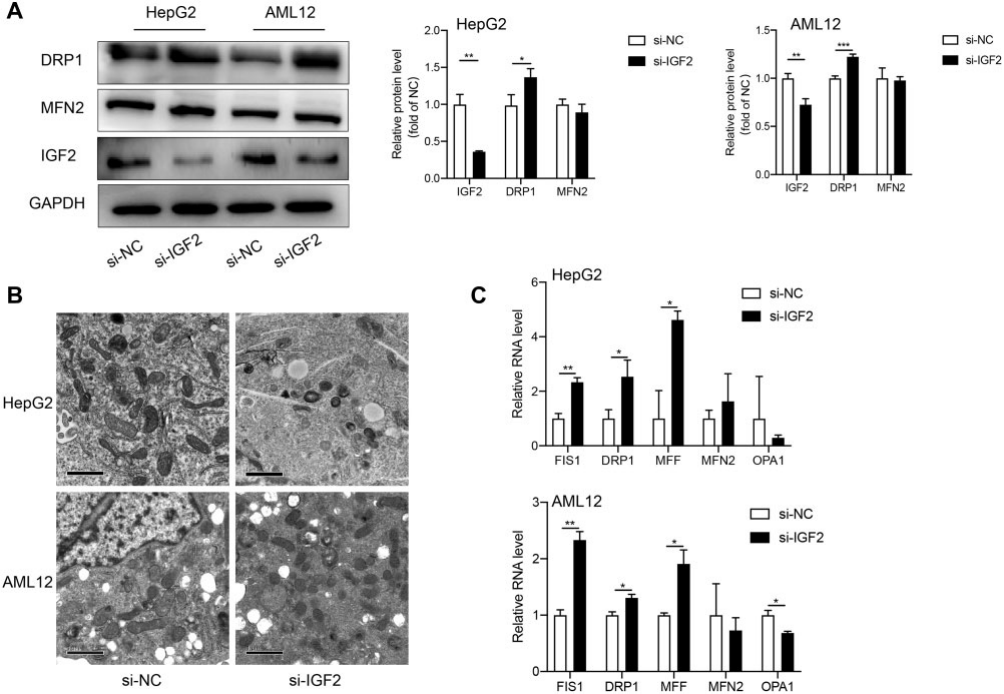
Effects of IGF2 knockdown on mitochondrial fission. (**A**) Western blotting analysis for protein levels of fission and fusion-related genes in HepG2 and AML12 cells following si-IGF2 treatment. Representative gel images (left) and the quantitative data (right) are presented. (**B**) Representative electron microscopic images for the mitochondria isolated from HepG2 and AML12 cells following IGF2 knockdown. Scale bar, 1 μm. (**C**) RT−qPCR analysis for relative expression of fission and fusion-related genes in HepG2 (upper) and AML12 (lower) cells following si-IGF2 treatment. Data were based on three independent experiments. Continuous variables were expressed as mean ± SD. ***P < 0.001, **P < 0.01, *P < 0.05.

To maintain the optimal functions, mitochondria display multiple characteristics including fusion, fission, and mitophagy (Mishra and Chan, 2016). Western blotting and RT–qPCR analyses revealed that IGF2 inhibition only increased the expression of dynamin-related protein 1 (DRP1), FIS1, and MFF, critical in regulating fission of mitochondria but had no significant effect on the expression of several fusion-related genes including MFN2 and OPA1 (Figure 5A and C). Accordingly, we speculated that IGF2 only regulates fission, but not fusion, of the mitochondria. Indeed, this hypothesis was validated by transmission electron microscopy, which revealed that si-IGF2-treated HepG2 and AML12 cells displayed smaller and fragmented mitochondria compared to si-NC-treated cells (Figure 5B). These findings demonstrate that IGF2 regulates mitochondrial fission.

### IGF2 replenishment partially rescues mitochondrial damages caused by IGF2 depletion

**Figure 6:**
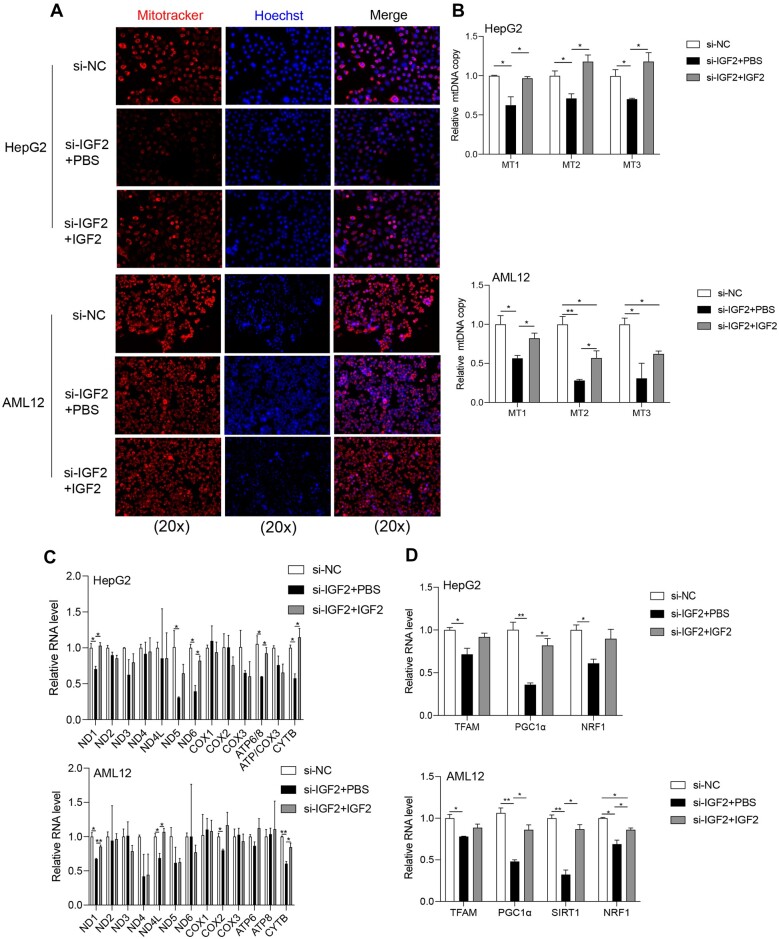
Replenishment of IGF2 partially rescues mitochondrial defects. HepG2 and AML12 cells were treated with si-IGF2 in the presence or absence of IGF2. (**A**) Representative images of mitotracker staining. (**B**) RT−qPCR analysis for relative mtDNA copy number. (**C**) RT−qPCR analysis for relative RNA levels of the mt-mRNAs. (**D**) RT−qPCR analysis for relative RNA levels of the mitochondrial transcription-related genes. Quantification was based on three independent experiments. Numbers were expressed as mean ± SD. **P < 0.01, *P < 0.05.

**Figure 7:**
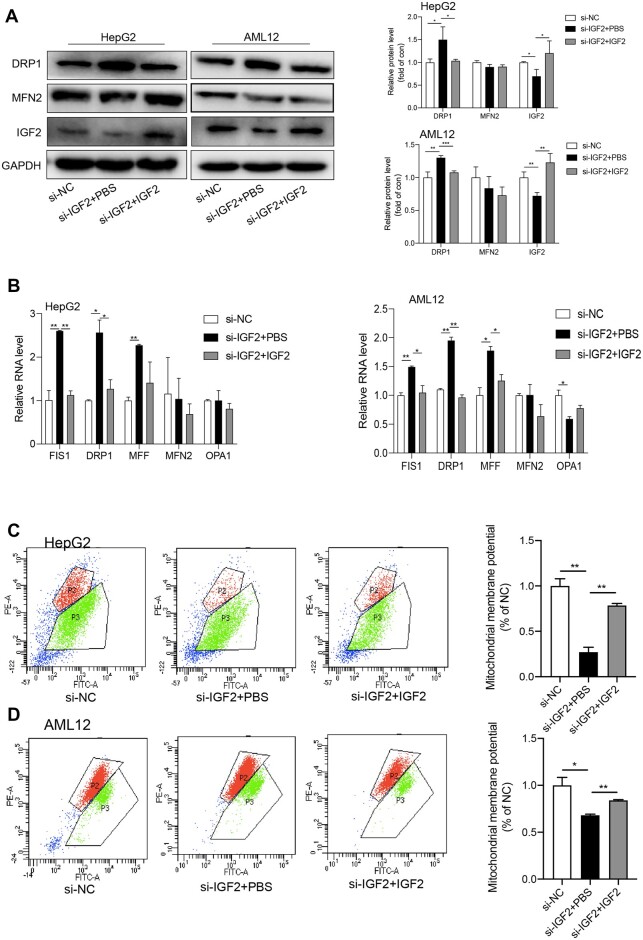
Replenishment of IGF2 partially rescues mitochondrial functions. HepG2 and AML12 cells were treated with si-IGF2 in the presence or absence of IGF2. (**A**) Western blotting analysis for protein levels of fission and fusion-related genes. Representative gel images (left) and the quantitative data (right) are presented. (**B**) RT−qPCR analysis for relative RNA levels of fission and fusion-related genes. (**C** and **D**) MMP was measured. Representative images of MMP detected by flow cytometry assays (left) and the MMP quantifications are presented. Quantification was based on three independent experiments. Numbers were expressed as mean ± SD. **P < 0.01, *P < 0.05.

To further confirm that the effects described above were caused by IGF2 depletion, we performed IGF2 replenishment experiments. As we have mentioned above, IGF2 depletion decreased mitochondrial contents as well as expression levels of mt-mRNAs and mitochondria-related genes. Not surprisingly, mitotracker staining confirmed that IGF2 replenishment increased mitochondrial contents in both HepG2 and AML12 cells after IGF2 silencing (Figure 6A). RT‒qPCR indicated that IGF2 replenishment increased mtDNA copy numbers, levels of several mt-mRNAs (e.g. ND1 and CYTB), and expression levels of mitochondrial genes (e.g. TFAM, PGC1α, and NRF1) in si-IGF2-treated HepG2 and AML12 cells (Figure 6B–D). Moreover, IGF2 replenishment decreased the protein and/or mRNA levels of DRP1, FIS1, and MFF in both HepG2 and AML12 cells treated with si-IGF2 (Figure 7A and B). Furthermore, IGF2 replenishment increased MMP that was decreased after IGF2 knockdown in HepG2 and AML12 cells (Figure 7C and D). Taken together, IGF2 depletion downregulated expression levels of PGC1α and SIRT1, increased ROS production and mitochondrial fission, and decreased mitochondrial biogenesis and ATP production, which contributed to the development of fatty liver and obesity. IGF2 replenishment could partially rescued mitochondrial damages caused by IGF2 depletion.

## Discussion

IGF2 is an imprint gene expressed as a paternal allele in most tissues. Its transcription is regulated by methylation of the differentially methylated region on the maternal allele. However, IGF2 is expressed biallelically in the liver, which explains the high level of the protein in circulation throughout an adult life. IGF2 is a hepatocyte mitogen that promotes liver repopulation. Changes in IGF2 expression have been implicated in the development of chronic liver diseases including simple steatosis, cirrhosis and even HCC. Although IGF2 is overexpressed in chronic liver diseases (Iizuka et al., 2002; Van Rooyen et al., 2011), the role of IGF2 in steatosis progression remains to be validated. Kessler et al. (2016) reported that transient overexpression of IGF2 in the liver induces free cholesterol accumulation and lipid droplet formation, which aggravates fatty liver disease. Mechanistically, IGF2 is thought to increase cholesterol synthesis *de novo*, given that IGF2 overexpression positively correlates with the synthesis of the key enzyme (HMG-CoAR) and gene (SREBF1) associated with cholesterol synthesis. Interestingly, a separate study found that inhibition of IGF2 induces hepatic steatosis in new-born mice, possibly from disrupted expression of nutrient metabolism-related genes (Lopez et al., 2018). This inconsistency may be attributed to the hyperbolic effects of IGF2 (Sandhu et al., 2003). Even so, the relationship between IGF2 and fatty liver as well as metabolism is only scarcely reported. To the best of our knowledge, this is the first report that systematically explores the relationship between IGF2 and mitochondrial functions in the liver.

In the present study, we revealed the upregulated expression of both IGF2 protein and mRNA in HepG2 and AML12 cells treated with FFA, liver tissues of HFD-induced obese mice, and serum of obese patients. Interestingly, we found that IGF2 mRNA was greatly upregulated by FFA treatment whereas the protein level only increased slightly. It was possible that FFA not only activated gene expression but also activated the negative regulation of IGF2 (Bergman et al., 2013). IGF2 underwent posttranslational processing via multiple cleavages. Moreover, IGF2 receptor (IGF2R) regulates the amount of circulating and tissue IGF2 by transporting the ligand into the cell and degrading it (Williams et al., 2012; Bach, 2018). In our study, AML12 and HepG2 cells were cultured with 0.75 mM FFA for 24 h to establish the *in vitro* hepatic steatosis model. It was proved to be a suitable and feasible method to stimulation simple steatosis (Gomez-Lechon et al., 2007; Moravcova et al., 2015). Moreover, it was believed that saturated fatty acid (palmitic acid) reduced the amount of both fully assembled OXPHOS complexes and complex subunits, decreased mtDNA-encoded subunits, and induced oxidative stress and caused mtDNA oxidative damage (Garcia-Ruiz et al., 2015). HepG2 cells treated with FFA exhibited high level of mitochondrial oxidative stress (Izdebska et al., 2017). Either palmitic or oleic acid was able to induce Ca^2+^-dependent swelling of mitochondria (Belosludtsev et al., 2014). Furthermore, bioinformatics analyses revealed that IGF2 participates in hepatic lipid metabolism and the regulation of mitochondrial functions, consistent with our hypothesis. Together, the role of IGF2 in regulating the pathogenesis of fatty liver seemed complicated. We then put our focus on the link between IGF2 and mitochondrial functions in fatty liver. We further found that IGF2 inhibition impaired mitochondrial functions, in particular biogenesis, disrupting mitochondrial dynamics of fusion and fission, respiration, and ROS production in hepatic cells. Finally, IGF2 replenishment partially rescued the aforementioned defects in mitochondria caused by IGF2 depletion.

IGF2 signals mainly via three receptors namely IGF1R, insulin receptor isoform A (IR-A), and IGF1R‒IR-A hybrid receptor (Livingstone, 2013). Although there is a certain degree of overlap in the functions of IGF2 and insulin, the two ligands induce different biological effects in target tissues. IGF2 mediates mitogenic signaling and survival in cancer, while insulin stimulates glucose uptake and metabolic activity (Nakae et al., 2001; Belfiore et al., 2009). IGF2 binding to IR-A or IGF1R recruits insulin receptor substrate protein, which binds to phosphatidylinositol 3-kinase and in turn activates protein kinase B (Pollak, 2008). IGF2R regulates the amount of circulating and tissue IGF2 by transporting the ligand into the cell and degrading it (Williams et al., 2012; Bach, 2018). Additionally, bioactivities of IGF2 are modulated by IGF-binding proteins (IGFBPs), which bind to IGFs but not insulin with high affinity. In general, IGFBPs limit the IGF access to IGF1R, thereby attenuating the bioactivities of these growth factors. These might be helpful to understand the basis for the different bioactivities of insulin and IGF2. Furthermore, IGF2 elicited more robust effects than insulin (Vella et al., 2019). IGF2 and insulin stimulated not only glycolytic activity but also mitochondrial biogenesis and activity as well as mitophagy. It appeared that the extents of activation by IGF2 and insulin were different. Expression levels of genes involved in mitochondrial biogenesis like PGC1α, PRC1, PNC1, NRF-2a, NRF1, TFAM, and MFN1 and genes involved in mitochondrial activity like NFE2L2, COX1, and cytoB were higher in breast cancer cells treated by IGF2 than that in cells treated by insulin. Hence, these might partially explain why insulin in the culture medium could not completely substitute IGF2 in the knockdown cell line for the regulation of mitochondrial functions in our research.

Numerous literatures show that mtDNA reflects the mitochondrial contents influenced by the balance between biogenesis and degradation in mitochondria (Williams, 1986; Wada and Nakatsuka, 2016; Bouchez and Devin, 2019). Research shows that PGC1α participates in the coactivation of nuclear genes encoding mitochondrial proteins that regulate mitochondrial contents and functions (Islam et al., 2020). Moreover, TFAM, a downstream target gene of PGC1α, regulates mtDNA transcription (Yakubovskaya et al., 2014). Interestingly, we found that IGF2 silencing modulated the expression levels of PGC1α and TFAM, implying that the low copy number of mtDNA in IGF2 knockdown cells was partially due to downregulation of these two genes. Accordingly, mtDNA encodes 13 mRNAs genes, which encompass the electron transfer chain (ETC) that regulates mitochondrial functions. ETC is the main process of oxygen consumption and ROS production in the cell (Aon et al., 2007; Yang et al., 2013). Multiple reports show that inhibiting ETC could increase ROS production and impair mitochondrial respiration (Erfurt et al., 2003; Aon et al., 2007). In this study, IGF2 silencing significantly disrupted multiple components of ETC, increased ROS production, and impaired mitochondrial basal and maximal respiration rates as well as ATP production in HepG2 and AML12 cells, underlining the close relationship between IGF2 and mitochondrial functions as previously described (Vella et al., 2019; Younis et al., 2020).

Mitochondrial fusion and fission processes maintain optimal number and desirable morphology of mitochondria (Skuratovskaia et al., 2020). Mitochondrial fission, which results in small and fragmented mitochondria, is regulated by several genes including DRP1, FIS1, and MFF, whereas mitochondrial fusion is mediated by OPA1 and MFN2 (Song et al., 2009; Loson et al., 2013; Varanita et al., 2015). IGF2 inhibition significantly enhanced mitochondrial fission and accumulation of small, fragmented mitochondria possibly regulated by overexpression of fission-related genes.

Notwithstanding, this study suffered several limitations. First, all our findings were based on *in vitro* models, and there is no guarantee that they can be replicated *in vivo*. Next, we only explored the relationship between IGF2 and energy metabolism under specific physiologic conditions. The role of IGF2 in the pathophysiology of steatosis was not evaluated.

In summary, steatosis upregulates IGF2 mRNA and protein levels in the liver tissues of obese mice. Bioinformatics analyses suggest that IGF2 regulates lipid metabolism and several mitochondrial functions. Consistently, inhibition of IGF2 in HepG2 and AML12 cells impairs mitochondrial respiration, decreases mitochondrial contents and MMP, promotes ROS production, and disrupts the balance between mitochondrial fission and fusion. Furthermore, we found that IGF2 performs its function by modulating the expression of PGC1α and TFAM, which in turn regulate the transcription of mitochondria and downstream ETC-related genes. Finally, IGF2 replenishment partially improves the damage in mitochondria caused by IGF2 depletion.

## Materials and methods

### Experimental animals

Our animal experiments were in accordance with the guidelines of the Animal Care Committee of Zhejiang University. Male 6-week-old C57BL/6J mice were purchased from the Model Animal Research Center of Nanjing University and were maintained on a 12-h light–darkness cycle. After a week of adaptive feeding, C57BL/6 mice were fed an HFD (35% carbohydrate, 20% protein, and 45% fat) for 3 months to induce obesity phenotype. Then, the livers of the mice were collected.

### Cell culture

HepG2 and AML12 cells (American Type Culture Collection) were cultured in Dulbecco's modified Eagle's medium (DMEM) supplemented with 10% fetal bovine serum and 100 IU/L penicillin‒streptomycin in a humidified incubator with 5% CO_2_ at 37°C. For induction of steatosis, the cells were treated with 0.75 mM FFA (oleic acid:palmitic acid, 2:1) for 24 h.

### Cell transfection

For transfection, HepG2 and AML12 cells were seeded in 6-well plates (10^5^ cells/ml) and cultured overnight. To knock down IGF2, small interfering RNAs (siRNAs) were used. In brief, to prepare siRNA transfection solution for each well, 10 μl 20 nM IGF2 siRNA or control siRNA (TSINGKE) was mixed with 100 μl OPTI-MEM reduced serum medium (Gibco). In parallel, 3 μl oligofectamine reagent (Invitrogen) was mixed with 100 μl OPTI-MEM. Following 5 min of incubation at room temperature, the two were mixed by gentle pipetting and incubated for 15 min at room temperature. Then, the mixture was added to the medium. Four hours after incubation in the humidified incubator with 5% CO_2_ at 37°C, the medium was replaced by the growth medium. In some cases, 100 ng/ml IGF2 (Sigma) was supplemented to the culture medium for 24 h before measurement as reported before (Pereira et al., 2019). After 48 h of transfection, RNA, DNA, and protein were extracted from the cells for further analyses. The sequences of IGF2 siRNAs were as follows: si-NC-F: GAAUUGCUCUCGGACAAUUCG; si-NC-R: CGAAUUGUCCGAGAGCAAUUC; si-hIGF2-1 (human)-F: UCGCCUCGUGCUGCAUUGCUGCUUA; si-hIGF2-1 (human)-R: UAAGCAGCAAUGCAGCACGAGGCGA; si-hIGF2-2 (human)-F: CUGGAGACGUACUGUGCUATT; si-hIGF2-2 (human)-R: UAGCACAGUACGUCUCCAGTT; si-mIGF2-1 (mouse)-F: GGAGCUUGUUGACACGCUUCA; si-mIGF2-1 (mouse)-R: UGAAGCGUGUCAACAAGCUCC; si-mIGF2-2 (mouse)-F: CUUGGACUUUGAGUCAAAUUGG; si-mIGF2-2 (mouse)-R: GGUCGUGCCAAUUACAUUUCA.

### Oil Red O staining

HepG2 and AML12 cells were first incubated for 24 h with FFA, washed twice with phosphate-buffered saline (PBS), fixed for 20 min with 4% formaldehyde, and thereafter rewashed three times with PBS. The cells were then stained for 15 min using Oil Red O (Nanjing Jiancheng Bioengineering Institute) and thereafter washed three times with PBS. The resultant lipid droplets were observed and photographed under an electron microscope (Zeiss).

### Nile red staining

HepG2 and AML12 cells were first pretreated with FFA as described above. After three washes using PBS, the cells were stained for 15 min after fixation with 4% formaldehyde using 0.05 μg/ml Nile red solution (Solarbio). The cell nuclei were stained using 496 diamidino-2-phenylindole (DAPI, Yeasen). The microscopic pictures were captured using an electron microscope (Zeiss).

### DNA, RNA, and RT‒qPCR analysis

Genomic DNA of the cells was extracted by using the DNA extraction kit (Solarbio), following the manufacturer’s instructions. Briefly, HepG2 and AML12 cells were centrifuged at 12000 rpm for 1 min after digestion with trypsin. RNA and proteins were digested using RNase A and protease K, respectively. The DNA was concentrated and isolated using the adsorption column. Total RNA was isolated from HepG2 and AML12 cells or mouse liver tissues using TRIzol (Solarbio). Serum total RNA was extracted using a miRNeasy kit (Qiagen). cDNA was synthesized from the RNAs by using reverse transcription and RT‒qPCR SYBR Green kits (Accurate Biotechnology). GAPDH was used as the internal control, with mRNA expression calculated based on the 2^−ΔΔCt^ formulae. The mtDNA copy number (the ratio of mitochondrial to nuclear DNA) was measured using RT‒qPCR. The various primer sets used in this study are shown in Supplementary Table S1. Liver tissues of HFD-fed mice and serum samples of obese patients were extracted as previously described (Xihua et al., 2019).

### Western blotting

HepG2 and AML12 cells were lysed *in situ* using 1× SDS-sample buffer (200 μl/well for 6-well plates) and thereafter denaturated. Protein samples were then fractionated on SDS–polyacrylamide gel before blotting. Primary antibodies used in this study were as follows: MFN2 (1:1000 dilution) (abclonal, A19678), DRP1 (1:1000 dilution) (abclonal, A2586), SIRT1 (1:1000 dilution) (Abcam, ab110304), PGC1α (1:1000 dilution) (Abclonal, A12348), OXPHOS (1:250 dilution) (Abcam, ab110413), IGF2 (1:250 dilution) (Santa Cruz, sc293176), and GAPDH (1:2000 dilution) (Abclonal, Ac001). GAPDH was used as the internal control. The protein bands were analyzed using Image J (NIH).

### Mitotracker staining

After 48-h transfection of si-IGF2, HepG2 and AML12 cells cultured in the 6-well plates were incubated in Mitotracker Red CMXRos solution at the concentration of 25 nM for 30 min in the humidified incubator with 5% CO_2_ at 37°C. Then, cell nucleus was stained with Hoechst. Finally, cellular morphology was visualized using the microscope (Zeiss).

### Transmission electron microscopy analysis

Fixed HepG2 and AML12 cells were washed with PBS, fixed with 1% buffered osmium tetroxide for 1 h, and then stained with aqueous 2% uranyl acetate. The samples were washed three times with water and dehydrated in increasing concentrations of ethanol (50%, 70%, 90%, and 100%). The samples were then cut into ultrathin 0.5-μm sections with a Leica UC7 ultramicrotome. A Hitachi H-7100 transmission electron microscope (Hitachi-High Technologies Co.) was used to analyze the stained sections.

### Mitochondrial respiration analysis

Mitochondrial respiration of HepG2 and AML12 cells was examined using the XF Mito Stress Test Kit (Seahorse Bioscience) on an XFe96 Extracellular Flux Analyzer in accordance with manufacturer’s instructions with minor adjustment. Shortly, the concentrations of oligomycin, carbonyl cyanide-4-(trifluoromethoxy)phenylhydrazone, and rotenone/antimycin used were 1.0, 1.0, and 0.5 μM, respectively. The respiration experiments were normalized to the number of cells.

### Cellular triglyceride measurement

Cellular triglyceride was measured using a triglyceride assay kit (Nanjing Jiancheng, A110). Following pretreatment of FFA for 24 h as described above, cells were lysed with standard RIPA lysis buffer and then centrifuged at 12000 *g* for 10 min at 4°C to collect the supernatant, which was subsequently used to detect the content of triglyceride. The cellular triglyceride was normalized to the protein concentration.

### MMP

MMP was detected using JC-1 assay kit (Solarbio) following its guidance. Simply, HepG2 and AML12 cells were incubated with JC-1 probes at 37°C for 20 min. Accumulation of JC-1 was determined using flow cytometry analysis (Becton Dickinson).

### ROS detection

To detect cellular ROS, an assay kit (Beyotime Institute of Biotechnology) was used according to the guidance of the manufacturer. Briefly, HepG2 and AML12 cells were incubated with dichlorofluorescein diacetate for 30 min at 37°C followed by detection of ROS using flow cytometry analysis (Becton Dickinson).

### Bioinformatics analysis

The gene expression profile of GSE116421, which is comprised of microarray-based gene expression profiles of three wild-type new-born mouse liver samples and three IGF2 knockout new-born mouse liver samples, was downloaded from the Gene Expression Omnibus (GEO) database (http://www.ncbi.nlm.nih.gov/geo/). Analysis of DEGs between x and y samples was performed using R package. The cut-off for differential expression threshold was based on |log2fold change (log2FC)| > 0.5 and *P*-value < 0.05. GO and KEGG pathway enrichment analyses were performed using the clusterProfiler package in R software.

### Statistical analysis

Continuous data were expressed as mean ± standard deviation (SD). Statistical significance was assessed using two-tailed Student's *t*-test at *P *<* *0.05. Each experiment was conducted in triplicate.

## Supplementary material

Supplementary material is available at *Journal of Molecular Cell Biology* online.

## Funding

This project was supported by grants from the Zhejiang Provincial Natural Science Foundation (LY20H070004) and the Zhejiang Provincial Medical Science and Technology Program (2020KY166 and 2018KY484).

**Conflict of interest**: none declared.

## Supplementary Material

mjab030_Supplementary_Data
