# Supplementary material for: Knockdown of insulin-like growth factor 2 gene disrupts mitochondrial functions in the liver
Source: J Mol Cell Biol. 2021 May 14;13(8):543–55. doi: 10.1093/jmcb/mjab030 (PMC8697341; doi:10.1093/jmcb/mjab030)
Supplement: mjab030_Supplementary_Data [file mjab030_supplementary_data.pdf]

Supplementary material

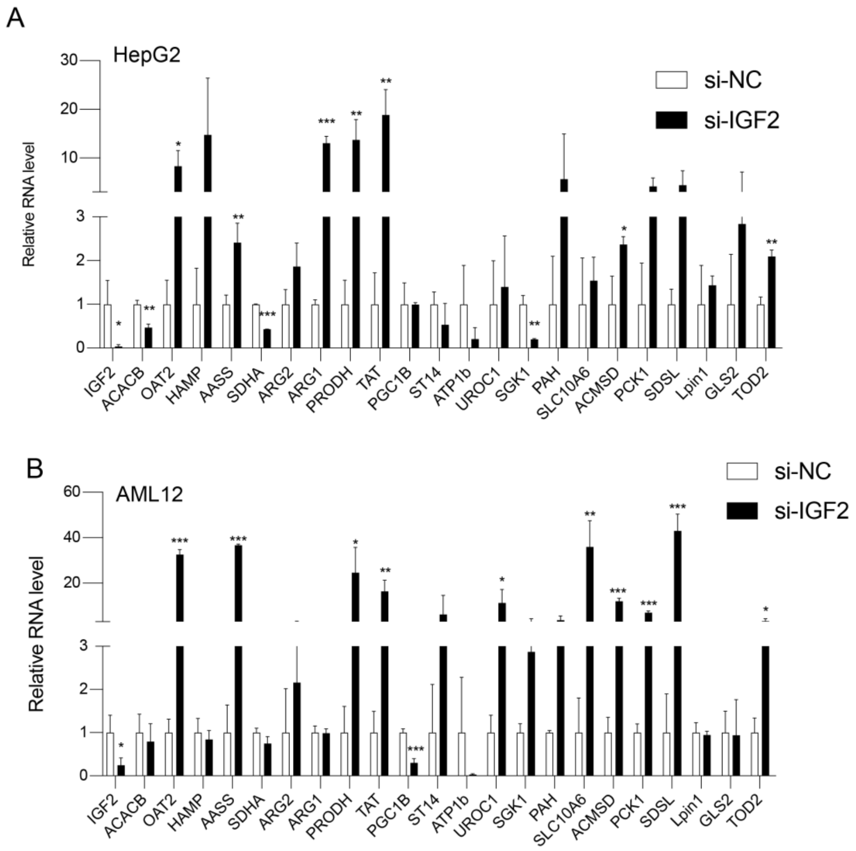

Supplementary Figure 1 Validation of mitochondria-related genes in HepG2 and AML12 cells. (**A**) RT-qPCR assays of mitochondria-related genes in HepG2 cells. (**B**) RT-qPCR assays of mitochondria-related genes in AML12 cells. Quantification was based on three independent experiments. Numbers were expressed as mean ± SD. ****P*<0.001, ***P* <0.01, **P* <0.05.

Supplementary Table 1 Sequences for primers used in this study.

	Sequences (5' to 3')	
	sense	anti-sense
**Human gene expression**		
IGF2	GACCGCGGCTTCTACTTCAG	AGAACTTGCCCACGGGGTAT
GAPDH	TGAACGGGAAGCTCACTGG	TCCACCACCCTGTTGCTGTA
ND1	CCACCTCTAGCCTAGCCGTTTA	GGGTCATGATGGCAGGAGTAAT
ND2	TGCAGGCACACTCATCACAG	GATGGCAGCTTCTGTGGAAC
ND3	TTCGACCCTATATCCCCCGC	GTAGGGCTCATGGTAGGGGTA
ND4	TTCCCCAACCTTTTCCTCCG	TGGATAAGTGGCGTTGGCTT
ND4L	TCGCTCACACCTCATATCCTC	AGGCGGCAAAGACTAGTATGG
ND5	GCTTAGGCGCTATCACCACT	TGCAGGAATGCTAGGTGTGG
ND6	CTCCCGAATCAACCCTGACC	TGGTGCTGTGGGTGAAAGAG
COX1	AATCATCGCTATCCCCACCG	CAGAGCACTGCAGCAGATCA
COX2	TGACCAGAGCAGGCAGATGAA	CCACAGCATCGATGTCACCATAG
COX3	ACCCTCCTACAAGCCTCAGA	TGACGTGAAGTCCGTGGAAG
ATP6/8	CCACCTACCTCCCTCACCAA	AATGATCAGTACTGCGGCGG
ATP/COX3	CCTAGAAATCGCTGTCGCCT	CTGTTAGGGGTCATGGGCTG
CYTB	AACTTCGGCTCACTCCTTGG	GATGAAAAGGCGGTTGAGGC
MT1	CACCCCTCACCCACTAGGATAC	TCCATGGGGACGAGAAGGATT
MT2	CCACCTACCTCCCTCACCAA	AATGATCAGTACTGCGGCGG
MT3	AATCATCGCTATCCCCACCG	CAGAGCACTGCAGCAGATCA
nDNA	TAGAGGGACAAGTGGCGTTC	CGCTGAGCCAGTCAGTGT
TFAM	CTCAGAACCCAGATGCAAA	GCCACTCCGCCCTATAA
PGC1a	TGAACTGAGGGACAGTGATTTC	CCCAAGGGTAGCTCAGTTTATC
NRF1	CTCTGAGAACTTCATGGAGGAAC	GAAGGCGAGTCTTCATCAGC
FIS1	GAACTACCGGCTCAAGGAATAC	CCCACGAGTCCATCTTTCTTC
DRP1	CGCAGAACCCTAGCTGTAATC	CTGGAATAACCCTTCCCATCAA
MFF	GGATGGATTGCACAGCCATT	GCGCCGACTCAGAGGTGT
MFN2	CCTTCCTTGAAGACACGTACAG	GATGCCTCTCACTTTGGATAGG
OPA1	GCATGCTAAAGGCACACCAAGTGA	TTCCCGCAGGCGAGGATAGTTATT
Tod2	GGAGAAGAAAATGAACTGCTACTTAAA	GGCTCTAAACCTGGAGTTCTTTC
SDHA	CCTTTCTGAGGCAGGGTTTA	AGAGCAGCATTGATTCCTCC

OAT2	AGCCTCCGTCAGCTATGTAAT	CATCGCCAGTCCCGTATCA
ARG2	GGTGGGTGAGGAAATCCAG	GTAGGAGAGGGCCACAAGG
ARG1	CCACCTAAGTAAATGTGGAAAC	ACCAAGAGGGAATTTGTAGAG
AASS	CGCTTCCTGCCTTTAGACCT	CCCCAAGTAAGCCCAACCAT
PRODH	CCGCCATGGCTCTGAGG	TTCGATGCAGCGCAAGAATG
TAT	F-TTTGGGACCCTGTACCATTGT	GCATTGGACTTGAGGAAGCTC
ST14	GGGACACACCCAGTATGGAGG	GAGGTTCTCGCAGGTGGTCTG
UROC1	F-CAGAGGCTCAGGGAAGCAAG	GAGCTGCACAGGGTAGTAGC
PAH	TCTTCTCCTCCCTAGTGCGA	CGGAAACCAGTGCAAGTCTG
ACMSD	AGGAGTAACAGTGCAAGCCC	TTTCACACAGCGCTCCATCT
SDSL	F-GTCTGGGATAGTTGGGCAGG	CGTCCATTCTGGAGACCAGG
PCK1	GTGGGGACGTTCAGAATCACA	ACCCAGATCCTGTCCCCTTT
Lpin1	CAATACAAAGGCGGCCACG	GAGCTCCTTCACGGTGACAA
GLS2	F-TGCGCTCCATGAAGGCTCT	TGCAGTCCAGTGGCCTTTAG
HAMP	GGGATGTGCTGCAAGACGTA	TTCTGGGGCAGCAGGAATAA
ACACB	TTGTGATGGTGACCCCCGAGGACCTTAAG	CGGGGATTCTCTTGGCAATGTCCACAATC
PGC1b	GCTGACAAGAAATAGGAGAGGC	TGAATTGGAATCGTAGTCAGTG
ATP1b	CTGCCTGCAGAGAGCCAG	ATTTCTTCCAGCTGCCCTCC
SGK1	GGCACCCTCACTTACTCCAG	GGCAATCTTCTGAATAAAGTCGTT
SLC10A6	CCTGTGTGCCTGACCATTCCT	CCAGGACCACACCAGCAA
**Mouse gene expression**		
IGF2	ACAACAGCTGACCTCATTTCC	TCGACTTCCCCACTGGGAT
GAPDH	ATGACATCAAGAAGGTGGTGAAGC	GAAGAGTGGGAGTTGCTGTTGAAG
ND1	GCTTTACGAGCCGTAGCCCA	GGGTCAGGCTGGCAGAAGTAA
ND2	CCTCCTGGCCATCGTACTCA	GAATGGGGCGAGGCCTAGTT
ND3	TAGTTGCATTCTGACTCCCCCA	GAGAATGGTAGACGTGCAGAGC
ND4	CTAGGCCATATGTGTTGGA	GTATATCGCCTCACACCTCA
ND4L	AGCTCCATACCAATCCCCATCAC	GGACGTAATCTGTTCCGTACGTGT
ND5	ACCAGCATTCCAGTCCTCAC	ATGGGTGTAATGCGGTGAAT
ND6	CTTGATGGTTTGGGAGATTGG	ACCCGCAAACAAAGATCACC
COX1	TCAACATGAAACCCCCAGCCA	GCGGCTAGCACTGGTAGTGA
COX2	ACCTGGTGAACTACGACTGCT	TCCTAGGGAGGGGACTGCTC
COX3	CCAAGGCCACCACACTCCTA	GGTCAGCAGCCTCCTAGATCA

ATP6	AGCTCACTTGCCCACTTCCT	AAGCCGGACTGCTAATGCCA
ATP8	AACATTCCCACTGGCACCTT	TCGTTCATTTTAATTCTCAAGGGGT
CYTB	AGACAAAGCCACCTTGACCCGAT	ACGATTGCTAGGGCCGCGAT
TFAM	GCCCGGCAGAGACGGTTAAA	GCCGAATCATCCTTTGCCTCC
PGC1a	GGACATGTGCAGCCAAGACTCT	CACTTCAATCCACCCAGAAAGCT
SIRT1	TGTGGTGAAGATCTATGGAGGC	TGTACTTGCTGCAGACGTGGTA
NRF1	TATGGCGGAAGTAATGAAAGACG	CAACGTAAGCTCTGCCTTGTT
FIS1	GCCTGGTTCGAAGCAAATAC	CACGGCCAGGTAGAAGACAT
DRP1	CGGTTCCCTAAACTTCACGA	GCACCATTTCATTTGTCACG
MFF	ATGCCAGTGTGATAATGCAAGT	CTCGGCTCTCTTCGCTTTG
MFN2	CTTGAAGACACCCACAGGAACA	GGCCAGCACTTCGCTGATAC
OPA1	GATGACACGCTCTCCAGTGAAG	CTCGGGGCTAACAGTACAACC
MT1	CCTATCACCCTTGCCATCAT	GAGGCTGTTGCTTGTGTGAC
MT2	ATCAGGATGAGCCTCAAACTCC	GATAAGAGGATAATGGCTATGGTTACTTC
MT3	CCCAGCTACTACCATCATTCAAGT	GATGGTTTGGGAGATTGGTTGATGT
nDNA	ATGGAAAGCCTGCCATCATG	TCCTTGTTGTTCAGCATCAC
Tod2	CTGGGGGATCCTCAGGCTAT	TGTCACTGTACTCGGCTGTG
OAT2	CAAGCAGGTTGACCGATCCT	CAGTTGGAAAGGCCCAAAGC
ARG2	CCAGCTGCCATTCGAGAAG	ATCATCTTGTGGGACATTAGTAAACTC
ARG1	GGAGAGCCTTCCTGCACTTT	GTGCCTTGGTCTACATTGAACATAC
AASS	CTGTCCGTGATGCTGGCTAT	CTGTCCGTGATGCTGGCTAT
PRODH	ACTTCACGTTGTGCAGGATGA	GTTGAGTGGAGTACGAGCGG
TAT	GTGCCCTGAAGAGCATCCTT	TCCGCAATTAACCGCTCTGT
ST14	F-GGAGGAGGCAGCCAATCTT	GAGCGAACTTGCACACGAAG
UROC1	ACAACTTGGACCCTGCTGTG	CAGTTGCTGAACACTTGCCC
PAH	GCGGTTTCCGTGAAGACAAC	ACGACAGTAAGCCAGCAACA
ACMSD	F-CCCTCGAAGGTTTGTGGGTT	CTAGCGCCTTAACACAACGC
SDSL	CTGGAGGACCAGGTCAACAC	GACGCCCCCATTGGGATTTA
PCK1	GGCAAGTGAAGACCTTGACAG	CTGACCACCTTACCTAAGAACGA
Lpin1	GACAGAGAATGGTCCCCCAG	CTTTTTGGTGTTGAGGGCCG
GLS2	AGTTCACCACGGCTCTGAAG	CACACCTGGATCCCAGACAC
HAMP	AGGGCAGACATTGCGATACC	GCAACAGATACCACACTGGGA
ACACB	CTACAAGACGGCGCAGGTCA	AGGCGCCAAACTTCAGCATC

PGC1b	GGTGTTCGGTGAGATTGTAGAG	GTGATAAAACCGTGCTTCTGG
ATP1b	CCTTCCCGGTGACTTTCCCTC	GCTGTAGCTGCACCACTCTG
SGK1	GCCCTGGGCTATCTGCACTC	TCCTCGGTAAACTCGGGATC
SLC10A6	TTACAGTGCTCTCGGCTGTG	GCTTCTGACTCTCCACAGAACA
